# Synergistic Triad of Mixed Reality, 3D Printing, and Navigation in Complex Craniomaxillofacial Reconstruction

**DOI:** 10.3390/bioengineering13010010

**Published:** 2025-12-23

**Authors:** Elijah Zhengyang Cai, Harry Ho Man Ng, Yujia Gao, Kee Yuan Ngiam, Catherine Tong How Lee, Thiam Chye Lim

**Affiliations:** 1 Division of Plastic, Reconstructive and Aesthetic Surgery, Department of Surgery, National University Hospital, Singapore 119228, Singapore; elijah_cai@nuhs.edu.sg (E.Z.C.); harry.hmng@gmail.com (H.H.M.N.); catherinethlee@yahoo.com (C.T.H.L.); 2 Division of Hepatobiliary and Pancreatic Surgery, Department of Surgery, National University Hospital, Singapore 119228, Singapore; yujiagao@nus.edu.sg; 3 Artificial intelligence Office, National University Health System, Singapore 119074, Singapore; surnky@nus.edu.sg; 4 Division of Thyroid and Endocrine Surgery, Department of Surgery, National University Hospital, Singapore 119228, Singapore; 5 Department of Biomedical Informatics, Yong Loo Lin School of Medicine, National University of Singapore, Singapore 119228, Singapore; 6 Department of Surgery, Yong Loo Lin School of Medicine, National University of Singapore, Singapore 119228, Singapore

**Keywords:** mixed reality (MR), intra-operative navigation, three-dimensional (3D) printing, Le Fort I osteotomy, craniofacial reconstruction, cleft lip and palate

## Abstract

The craniofacial skeleton is a complex three-dimensional structure, and major reconstructive cases remain challenging. We describe a synergistic approach combining intra-operative navigation, three-dimensionally (3D) printed skull models, and mixed reality (MR) to improve predictability in surgical outcomes. A patient with previously repaired bilateral cleft lip and palate, significant midfacial retrusion, and a large maxillary alveolar gap underwent segmental Le Fort I osteotomy and advancement. Preoperative virtual planning was performed, and reference templates were uploaded onto MR glasses. Intra-operatively, the MR glasses projected the templates as holograms onto the patient’s skull, guiding osteotomy line marking and validating bony segment movement, which was confirmed with conventional navigation. The 3D-printed skull model facilitated dissection and removal of intervening bony spicules. Preoperative planning proceeded seamlessly across software platforms. Osteotomy lines marked with MR showed good concordance with conventional navigation, and final segment positioning was accurately validated. Postoperative outcomes were satisfactory, with re-established occlusion and closure of the maxillary alveolar gap. The combined use of conventional navigation, 3D-printed models, and MR is feasible and allows safe integration of MR into complex craniofacial reconstruction while further validation of the technology is ongoing.

## 1. Introduction

The craniomaxillofacial (CMF) skeleton is a complex three-dimensional (3D) structure. It includes the bones of the skull and face, including the upper jaw (maxilla), lower jaw (mandible), cheekbones, nasal bones, and surrounding structures. Together, these bones support vital functions such as breathing, eating, speech, and vision, while also providing the underlying framework that determines facial appearance. Because of their intricate anatomy and close relationship to the brain, eyes, and airway, reconstruction of this region requires both precision and careful planning [1]. It is also the main aesthetic unit of the body, with significant psychosocial implications if pathology affects it [2,3].

Pathologies that affect the CMF skeleton include trauma, tumors, and congenital malformations [4]. The reconstructive surgeon’s goal is to restore normal craniofacial anatomy. Patient-reported outcomes following CMF skeleton reconstruction reflect patients’ desires in the restoration of esthetic beauty, and underscore the need for accurate surgical execution [5]. Complex cases are challenging, as a considerable amount of subjectivity is involved when assessing the magnitude and direction of bone segment movement intra-operatively during bony reconstruction [6,7,8].

Adjuncts have been widely adopted to allow for greater predictability and accuracy. These include intra-operative navigation [7,9,10,11] and 3D-printed skull models [12,13,14,15,16]. Conventional navigation devices involve a surgical probe, a localizer, and a display monitor. Surgeons use the probe to point at a particular location on the patient’s skull, which is then displayed on computed tomography (CT) scan images. This provides surgeons with real-time visual guidance during the procedure. Three-dimensional skull models allow surgeons to gain a greater appreciation of the patient’s anatomy, perform preoperative surgical simulation, and serve as an intra-operative ‘3D road map’ during dissection [17,18].

Mixed reality (MR) has emerged as a potential tool for intra-operative navigation [19,20,21,22]. The surgeon dons MR glasses, and a skull reference hologram is projected onto the operative field. Using an automated registration tool, the hologram can be projected directly onto the patient’s physical skull using predetermined bony landmarks. This is akin to the surgeon having ‘X-ray eyes,’ enabling them to see through the patient’s anatomy. The reference hologram acts as a template to guide surgeons during dissection, osteotomy planning, and validation of bony segment movement.

MR is still in its early stages of adoption for craniofacial reconstruction [19,20,22]. Further validation and developments in both hardware and software are required. At its current stage, it should still be used in conjunction with other validated adjuncts such as conventional navigation devices and 3D-printed skull models. The existing literature lacks details on how this could be performed.

This paper aims to provide a detailed, step-by-step technical guide for the integrated use of these three technologies, using a complex clinical case as an exemplar, to facilitate safer adoption for novice MR users. We illustrate this through a complex case of bilateral cleft lip and palate, in which the patient presented with underlying skeletal deformities of the upper jaw requiring reconstructive orthognathic surgery.

## 2. Materials and Methods

**Figure 1:**
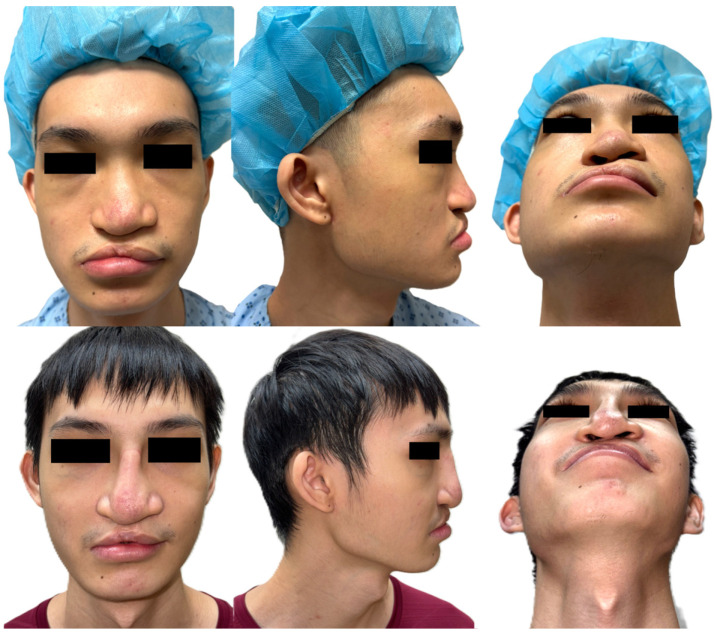
(**Top**)—Pre-operative images of a patient with previously repaired bilateral cleft lip and palate. Maxillary retrusion that is worse on the right and associated cleft nasal deformity. (**Bottom**)—Postoperative images demonstrating improvement in maxillary projection after segmental Lefort 1 osteotomy and advancement. A nasal rib cartilage spacer graft was also inserted, in preparation for a staged formal septorhinoplasty in future.

**Figure 2:**
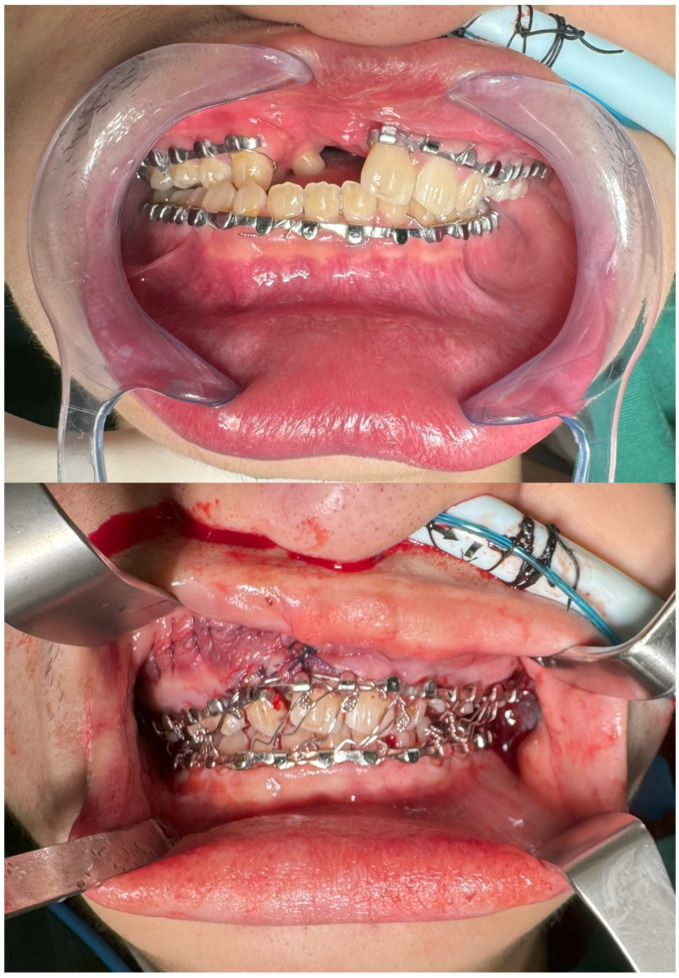
(**Top**)—Pre-operative occlusion, demonstrating large maxillary alveolar arch gap. (**Bottom**)—Postoperative occlusion, demonstrating restoration of occlusion and closure of maxillary alveolar arch gap.

The patient was a 21-year-old Chinese male with a history of bilateral cleft lip and palate that had been previously repaired (Figure 1). He presented with significant facial deformity, malocclusion, and midfacial retrusion, more pronounced on the right. Additional findings included a maxillary alveolar gap, mal-aligned dental arches with skeletal crossbite on the lesser segment on the right (Figure 2), and a canted occlusal plane of the larger segment of the maxillary alveolar bone on the left, with severe dental arch deviation of 11 mm to his left (reference taken from his upper and lower central incisors). The patient underwent preoperative orthodontic treatment before a segmental Le Fort I osteotomy. The surgical goals were closure of the maxillary arch gap, restoration of occlusion, and improved midfacial projection. A nasal dorsal spacer graft was inserted to facilitate subsequent definitive septorhinoplasty.

### 2.1. Preoperative Planning

A non-contrast computed tomography (CT) scan of the face (Image-Guided Surgery Protocol: gantry tilt 0°, 1 mm cuts) was performed, and Digital Imaging and Communications in Medicine (DICOM) data were obtained. CBCT/CT measurement methods were performed in line with recent CBCT morphometric protocols [23].

Virtual planning was performed in three stages.

**Figure 3:**
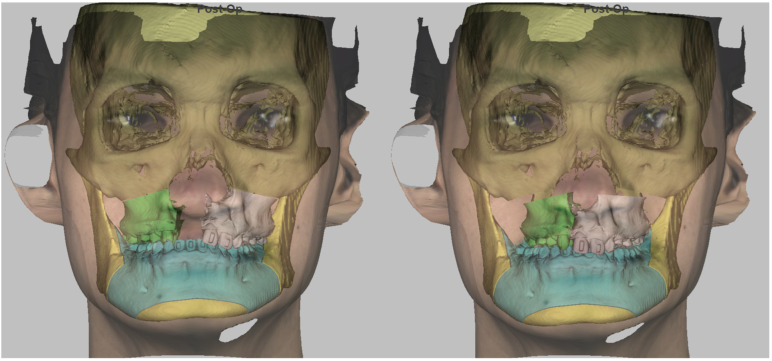
Pre-operative planning (stage 1): Determining lines of osteotomy and bony segments for reconstruction. (**Left**)—Bony segments of left and right maxilla prior to movement. (**Right**)—Bony segments of left and right maxilla after simulated movement.

First, osteotomy lines and movement vectors were defined using Dolphin 3D^®^ (Patterson Dental Holdings Inc, Chatsworth, CA, USA). Two maxillary bony segments were marked, and their planned movements allowed restoration of normal occlusion (Figure 3).

**Figure 4:**
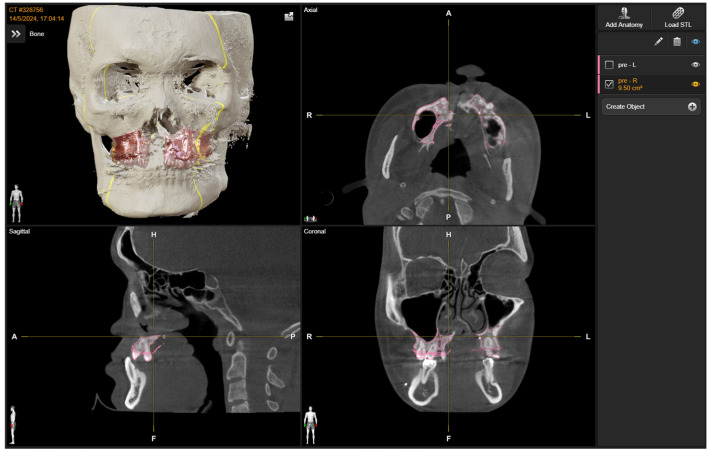
Pre-operative planning (stage 2): Creation of pre-operative reference templates (MR Template 1 in red) for intra-operative navigation.

Second, intra-operative navigation templates were generated using Brainlab Elements Contouring^®^ 4.0 (Brainlab^®^, Munich, Germany) contained the two maxillary segments before osteotomy, guiding osteotomy marking. MR Template 2 contained the segments after osteotomy, movement, and fusion, serving to validate final positioning (Figure 4). DICOM data were converted into STL files for projection onto the MR system.

**Figure 5:**
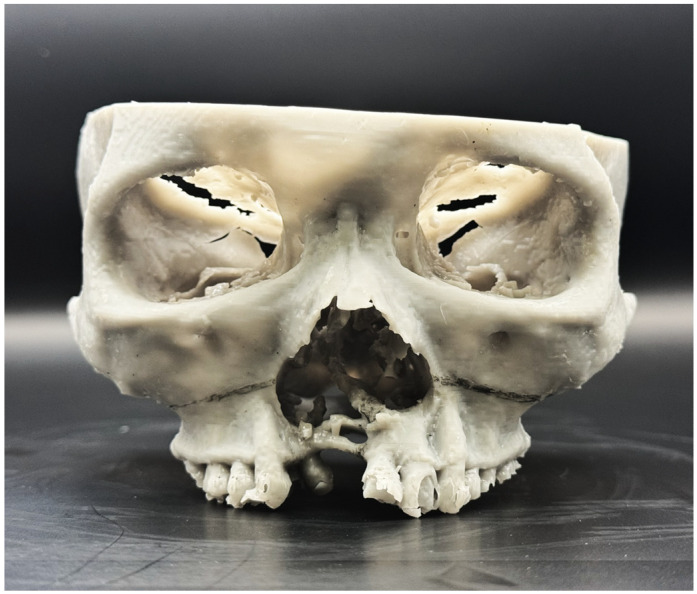
Pre-operative planning (stage 3): 3D-printed skull model with proposed osteotomy lines marked.

Third, a 3D-printed skull model was created using Chitubox^®^ Basic 2.3 software (Shenzhen CBD Technology Co., Ltd., Shenzhen, China) and a resin-based printer (3D3^®^ Max5, Star3D Material Development Company, Singapore, Singapore) (Figure 5). The model aided in anatomical appreciation, osteotomy planning, and patient counseling.

### 2.2. Intra-Operative Navigation

A standard segmental Le Fort I osteotomy was performed under general anesthesia. Bilateral upper buccal sulcus incisions were made, and dissection exposed the maxillae. Two registration markers were added at the crown of the incisors, and another two at the inferior border of the pyriform aperture. The 3D model also provided dissection guidance, particularly in removing intervening bone spicules extending into the pyriform aperture and anterior nasal cavity, while preserving nasal integrity.

**Figure 6:**
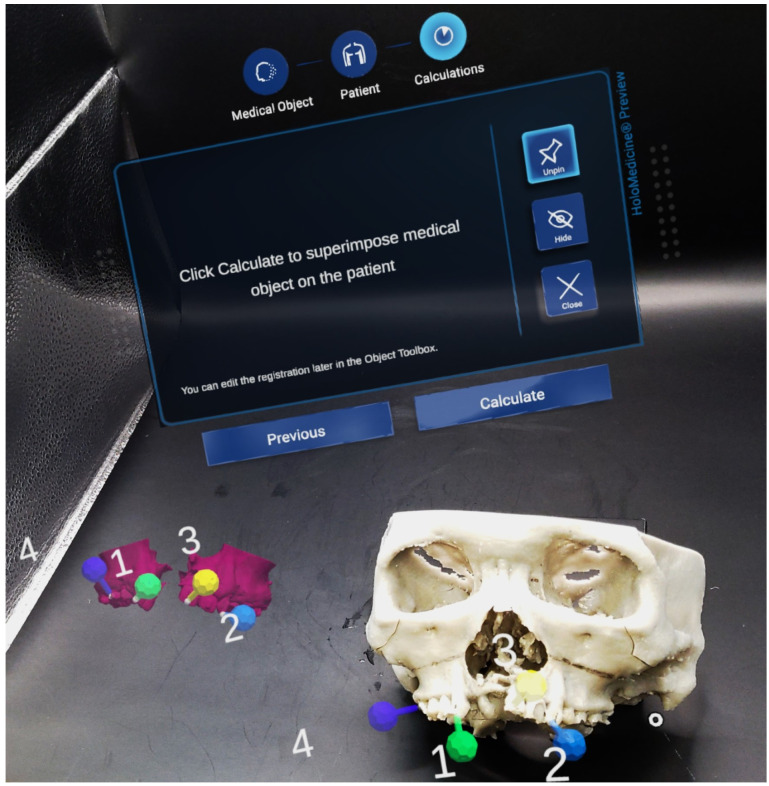
Markers placed on skull hologram (**left**) with corresponding markers on 3D-printed skull model (**right**). Once markers were placed, automatic superimposition of hologram onto the skull model could take place.

**Figure 7:**
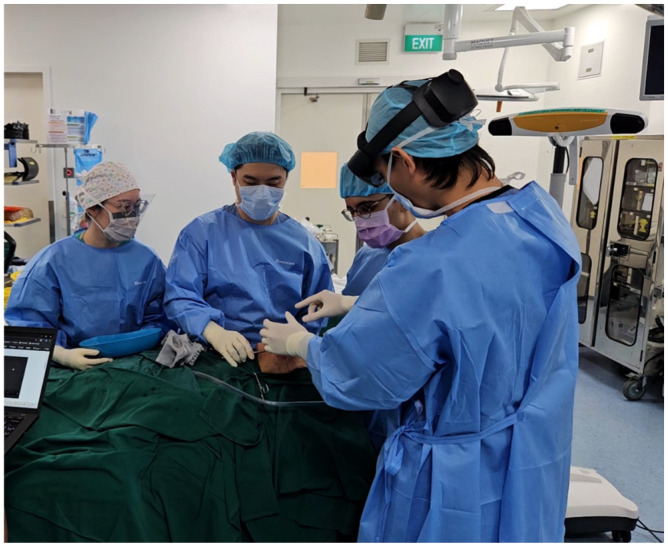
Intra-operative setup. Surgeon has donned mixed-reality glasses.

**Figure 8:**
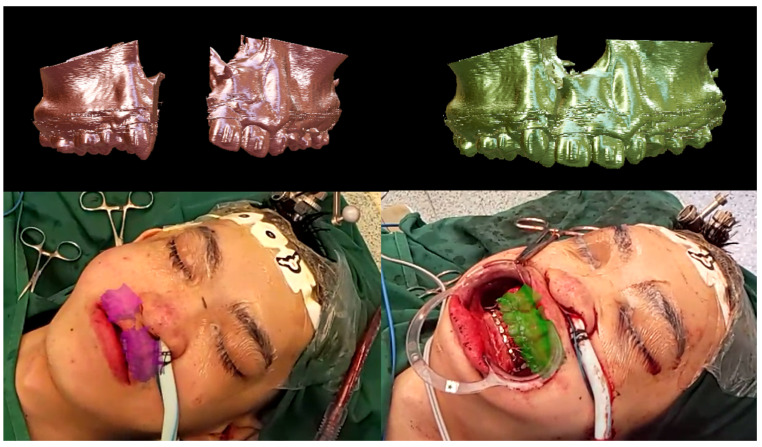
Application of intra-operative navigation using MR. (**Top left**)—MR Template 1, bony segments prior to segmental movement. (**Top right**) —MR Template 2, bony segments after segmental osteotomy and movement. (**Bottom left**) —First-person view of surgeon donning MR glasses, demonstrating superimposed hologram (MR Template 1). The hologram guides the surgeon with marking of osteotomy lines; (**Bottom right**) —First-person view of surgeon donning MR glasses, demonstrating superimposed hologram (MR Template 2). This is performed after segmental osteotomy and movement of the bony segments. The hologram guides the surgeon in validating the final position of the bony segments.

Mixed-reality guidance was then used. The surgeon donned HoloLens 2^®^ glasses (Microsoft^®^, Redmond, WA, USA) with Virtual Surgery Intelligence^®^ software (apoQlar^®^, Hamburg, Germany) (Figure 6). MR Template 1 was projected and registered automatically to the skull (Figure 7 and Figure 8, above). Osteotomy lines were marked and verified against the preoperative plan using a navigation probe (Kick^®^ 2 Navigation Station, Brainlab^®^, Munich, Germany).

**Figure 9:**
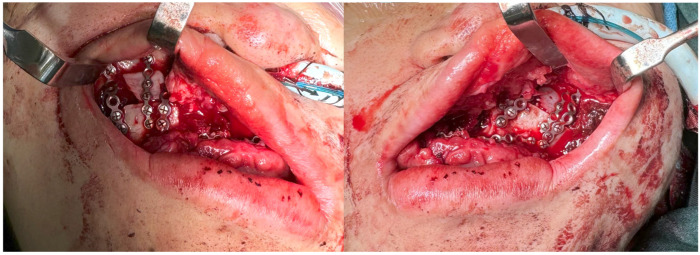
Once the maxillary bony segments have been moved into their final position, fixation was performed with titanium plates and screws.

Osteotomies were performed with an oscillating saw and osteotomes, followed by maxillary down-fracture. A prefabricated wafer guided repositioning of the freed segments. MR Template 2 was projected (Figure 8, bottom), and registration confirmed segment alignment with the hologram, which was further validated using conventional navigation. Titanium plates and screws (DePuy Synthes^®^, Raynham, MA, USA) secured fixation (Figure 9). Hemostasis was achieved and wounds closed.

## 3. Results

**Figure 10:**
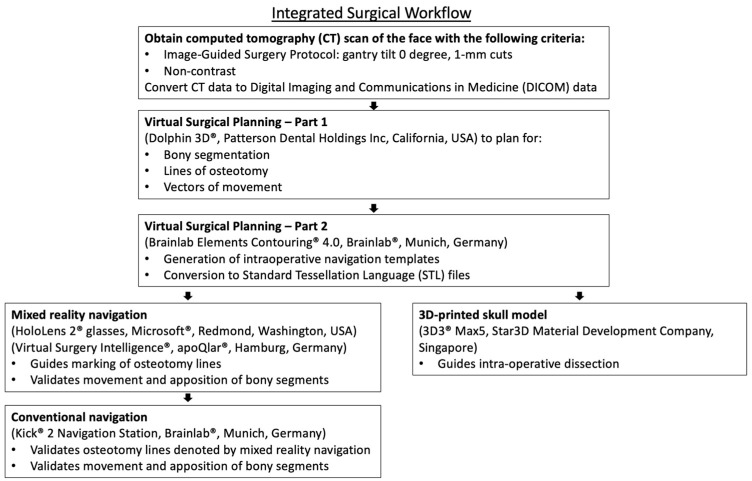
Integrated surgical workflow outlining preparation, file conversion process, and intra-operative usage of all 3 technologies.

Preoperative planning was seamless between the software platforms. Planning time was about 30 min, which included the conversion and uploading of files. Image data files were easily transferred in DICOM and STL formats, with the final STL file uploaded to the MR glasses. Virtual planning proceeded in the same manner as for conventional navigation and 3D printing, with no additional steps required beyond uploading the STL file (Figure 10).

Intra-operative usage was straightforward. There was an additional MR registration time of 10 min and extra operative time of 15 min. The user interface was intuitive, and automatic registration required surgeons to place four virtual markers on the skull hologram and four corresponding markers on the patient’s skull. The software then superimposed the hologram on the patient’s anatomy. Opacity adjustment allowed simultaneous visualization of the hologram (MR Template 1) and the underlying skull. Osteotomy lines marked with MR demonstrated good concordance (1–2 mm deviation) with conventional navigation, enabling more accurate marking. After osteotomy and mobilization, MR Template 2 was projected to validate segment positioning. Concordance (1–2 mm deviation) was again observed between MR guidance and conventional navigation.

The operative time was 10 h and 50 min. The patient was managed in the intensive care unit until stable. The patient was brought into operating theater at postoperative day 5 for washout and closure due to collections seen in the CT-scan. Patient was discharged well and stable.

The postoperative outcomes were consistent with the preoperative plan. The occlusal plane was corrected and the maxillary alveolar arch midline was established with the closure of the large alveolar gap (Figure 2, bottom). Midfacial projection improved, and nasal dorsal height was enhanced with a rib cartilage spacer graft (Figure 1, bottom). In a recent review 1 year from surgery, it was established that the patient is currently satisfied regarding his symmetry and postoperative changes. The patient is scheduled for definitive septorhinoplasty at a later stage.

## 4. Discussion

Craniofacial reconstruction can be challenging. Conventional surgical techniques without adjuncts often rely on subjective intra-operative assessment by the surgeon. This can often lead to inaccuracies and undesirable clinical outcomes, especially with complex cases [6,10,24]. Adjuncts such as intra-operative navigation and 3D-printed skull models provide surgeons with objective methods of intra-operative evaluation, achieving more predictable results.

Conventional intra-operative navigation has been shown to improve surgical outcomes [9]. The major components of a navigation system include a surgical probe, a localizer and a display monitor [17,25]. A skull reference array has to be anchored onto the patient’s skull via a small 1 cm incision in the patient’s scalp. Intra-operative navigation allows surgeons to pin-point exactly where a particular anatomical location is physically on the patient’s skull, which would be displayed on a corresponding point on the patient’s computed tomography (CT) scan on the display monitor. A margin of error of up to 1 to 2 mm could be achieved with infra-red based localizers [26]. Conventional navigation allows surgeons to determine immediately during the surgery if the desired movement of bone segments has been achieved.

Conventional navigation has its limitations. These include a high capital cost, the need for bulky equipment, and a steep learning curve. An additional incision over the scalp is required for the insertion of a reference array for bone-based registration. There is a need for multiple registration points, which might not always be present, especially in cases of severe facial fractures. Without an adequate number of robust registration points, the accuracy of navigation would be compromised. The surgeon is not able to maintain continuous line-of-sight with the surgical field. Frequent breaks in line-of-sight are required when surgeons refer to the display monitor.

A 3D-printed skull model is another adjunct to aid with complex cases [14]. A 3D-printed skull model provides surgeons with a tool for pre-operative planning [18,27,28]. The trajectory of dissection can be visualized and regions of osteotomy marked. Intra-operatively, it provides surgeons with a visual and tactile guide, akin to a road-map. For cases that require implant reconstruction, a 3D model serves as a template for pre-bending of implants prior to insertion.

A 3D-printed skull model has its limitations. Despite the rise in number of 3D printer brands in the market, it can still be costly for certain centers to acquire a suitable printer. There is a learning curve involved in converting DICOM data into a printable STL file format. Turnover duration can be significant if printing is outsourced, ranging from 5 to 21 days. In-house printing shortens the process, with prints ready within 8 to 24 h, as is the case in our institution, where the 3D printing capability is housed in our operation theater suites. Intra-operatively, a skull model provides a surgical road-map, but does not allow validation of bony movements made after reconstruction.

Mixed reality (MR) within the realm of craniofacial reconstruction has been described as a tool for patient education, surgical training and simulation, pre-operative planning, and intra-operative navigation [29,30,31]. One of its greatest utilities is the ability to use it as an intra-operative navigation tool. It is akin to granting surgeons ‘X-ray eyes’, the ability to see through the patient’s anatomy. The surgeon dons MR glasses, and is able to see the skeletal anatomy. Lines of osteotomy can be marked out based on pre-operative plans. Immediate validation of the accuracy of bony movements can be performed. The procedure for the localization of osteotomies is hands-free, unlike conventional navigation where a probe has to be manipulated by the surgeon. The surgeon maintains continuous line-of-sight over the surgical field, without having to refer to the display monitor.

The challenge comes with implementing MR as a navigation tool. Significant improvements are still required before it can function independently as an adjunct [30,32,33]. The registration process still requires further development and validation with larger clinical studies. Parallax error can occur when viewing the skull hologram from extremes of gaze. The hologram does not provide tactile guidance during osteotomy, and the trajectories of the oscillating saw and chisel still have to be determined with a considerable amount of subjectivity. This is in contrast to conventional cutting guides, where a localized physical barrier is formed to guide the cutting instruments according to pre-planned vectors. The opacity of the hologram at times obscures the operative field, and the opacity has to be adjusted accordingly.

We advocate combining conventional intra-operative navigation, 3D-printed skull models and cutting guides to allow MR to be utilized safely. These validated adjuncts could be used as a basis for comparison and validation of MR in craniofacial reconstruction. The described approach serves as a guide for surgeons and researchers looking to adopt MR for research and clinical practice in complex craniofacial reconstructive procedures. We hope that with wider adoption of MR, this could lead to greater user experience and feedback, which would contribute to further development of the technology.

There are limitations in the design of this study. This is a single case report that demonstrates a workflow, and not a comparative study. Further studies and development of both hardware and software are required for MR before it can be implemented as the sole adjunct for intra-operative navigation. A validated automatic registration process is required to allow the hologram to overlay precisely on the patient’s physical skull. A self-correcting mechanism based on an improved Light Detection and Ranging (LiDAR) system to mitigate the issue of parallax error would improve precision of visualization [34]. Haptic gloves or sensors attached to bone cutting instruments could provide surgeons with feedback required to perform osteotomies in the correct vector [35]. A visual feedback system with holographic visual cues, like visual alerts or color changes in the bone segments, as well as collision control will also be helpful. An adaptive system that automatically adjusts the opacity of the holograms, based on ambient lighting and angles of gaze, would further enhance the MR experience. Integrating artificial intelligence would allow the MR system to identify critical structures to preserve during dissection, providing surgeons with an early warning system [36].

## 5. Conclusions

MR navigation is a promising tool that can be added to the armamentarium of clinical adjuncts in complex craniomaxillofacial reconstruction. However, further hardware and software development is required. This case report showed that a combination of conventional navigation, 3D printing, and MR is suitable until MR can be validated as a sole navigation adjunct. Nonetheless, MR has the potential to replace the bulky equipment associated with conventional navigation with a lightweight, wearable headset that is more accessible to surgeons.

## Data Availability

The data is contained within the article.

## Abbreviations

The following abbreviations are used in this manuscript:

CMF	Craniomaxillofacial
3D	Three-dimensional
CT	Computed tomography
DICOM	Digital Imaging and Communications in Medicine
MR	Mixed reality
STL	Standard Tessellation Language
LiDAR	Light Detection and Ranging

## References

[1] Anderson B.W., Kortz M.W., Al Kharazi K. (2018). Anatomy, Head and Neck, Skull.

[2] Dissaux C., Diop V., Wagner D., Talmant J.-C., Morand B., Bruant-Rodier C., Ruffenach L., Grollemund B. (2021). Aesthetic and Psychosocial Impact of Dentofacial Appearance after Primary Rhinoplasty for Cleft Lip and Palate. J. Cranio-Maxillofac. Surg..

[3] Du H., Liang H., Qi Z., Jin X. (2024). A Prospective Investigation of Patient Satisfaction and Psychosocial Status Following Facial Bone Contouring Surgery Using the FACE-Q. Aesthetic Plast. Surg..

[4] Hunt J.A., Hobar C.P. (2003). Common Craniofacial Anomalies: Conditions of Craniofacial Atrophy/Hypoplasia and Neoplasia. Plast. Reconstr. Surg..

[5] Almasri A.M., Hajeer M.Y., Sultan K., Aljabban O., Zakaria A.S., Alhaffar J.B., Almasri A., Hajeer M.Y., Zakaria A.S. (2024). Evaluation of Satisfaction Levels Following Orthognathic Treatment in Adult Patients: A Systematic Review. Cureus.

[6] Troise S., De Fazio G.R., Committeri U., Spinelli R., Nocera M., Carraturo E., Salzano G., Arena A., Abbate V., Bonavolonta P. (2025). Mandibular Reconstruction after Post-Traumatic Complex Fracture: Comparison Analysis between Traditional and Virtually Planned Surgery. J. Stomatol. Oral Maxillofac. Surg..

[7] Raveggi E., Gerbino G., Autorino U., Novaresio A., Ramieri G., Zavattero E. (2023). Accuracy of Intraoperative Navigation for Orbital Fracture Repair: A Retrospective Morphometric Analysis. J. Cranio-Maxillofac. Surg..

[8] Alfayez E. (2025). Current Trends and Innovations in Oral and Maxillofacial Reconstruction. Med. Sci. Monit. Int. Med. J. Exp. Clin. Res..

[9] Cai E.Z., Koh Y.P., Hing E.C.H., Low J.R., Shen J.Y., Wong H.C., Sundar G., Lim T.C. (2012). Computer-Assisted Navigational Surgery Improves Outcomes in Orbital Reconstructive Surgery. J. Craniofac. Surg..

[10] Klug C., Schicho K., Ploder O., Yerit K., Watzinger F., Ewers R., Baumann A., Wagner A. (2006). Point-to-Point Computer-Assisted Navigation for Precise Transfer of Planned Zygoma Osteotomies from the Stereolithographic Model into Reality. J. Oral Maxillofac. Surg. Off. J. Am. Assoc. Oral Maxillofac. Surg..

[11] Anand M., Panwar S. (2021). Role of Navigation in Oral and Maxillofacial Surgery: A Surgeon’s Perspectives. Clin. Cosmet. Investig. Dent..

[12] Kang S.-H., Kim M.-K., You T.-K., Lee J.-Y. (2015). Modification of Planned Postoperative Occlusion in Orthognathic Surgery, Based on Computer-Aided Design/Computer-Aided Manufacturing–Engineered Preoperative Surgical Simulation. J. Oral Maxillofac. Surg..

[13] Cohen A., Laviv A., Berman P., Nashef R., Abu-Tair J. (2009). Mandibular Reconstruction Using Stereolithographic 3-Dimensional Printing Modeling Technology. Oral Surg. Oral Med. Oral Pathol. Oral Radiol. Endodontology.

[14] Ghai S., Sharma Y., Jain N., Satpathy M., Pillai A.K. (2018). Use of 3-D Printing Technologies in Craniomaxillofacial Surgery: A Review. Oral Maxillofac. Surg..

[15] Tack P., Victor J., Gemmel P., Annemans L. (2016). 3D-Printing Techniques in a Medical Setting: A Systematic Literature Review. Biomed. Eng. Online.

[16] Sarkarat F., Tofighi O., Jamilian A., Fateh A., Abbaszadeh F. (2023). Are Virtually Designed 3D Printed Surgical Splints Accurate Enough for Maxillary Reposition as an Intermediate Orthognathic Surgical Guide. J. Maxillofac. Oral Surg..

[17] Arboit L., Tel A. (2025). Software Used for Virtual Surgical Planning. Atlas of Virtual Surgical Planning and 3D Printing for Cranio-Maxillo-Facial Surgery.

[18] Mayo W., Mohamad A.H., Zazo H., Zazo A., Alhashemi M., Meslmany A., Haddad B. (2022). Facial Defects Reconstruction by Titanium Mesh Bending Using 3D Printing Technology: A Report of Two Cases. Ann. Med. Surg..

[19] Cai E.Z., Gao Y., Ngiam K.Y., Lim T.C. (2021). Mixed Reality Intraoperative Navigation in Craniomaxillofacial Surgery. Plast. Reconstr. Surg..

[20] Cai E.Z., Yee T.H., Gao Y., Lu W.W., Lim T.C. (2024). Mixed Reality Guided Advancement Osteotomies in Congenital Craniofacial Malformations. J. Plast. Reconstr. Aesthet. Surg..

[21] McJunkin J.L., Jiramongkolchai P., Chung W., Southworth M., Durakovic N., Buchman C.A., Silva J.R. (2018). Development of a Mixed Reality Platform for Lateral Skull Base Anatomy. Otol. Neurotol..

[22] Ackermann J., Liebmann F., Hoch A., Snedeker J.G., Farshad M., Rahm S., Zingg P.O., Fürnstahl P. (2021). Augmented Reality Based Surgical Navigation of Complex Pelvic Osteotomies—A Feasibility Study on Cadavers. Appl. Sci..

[23] Azizia T., Sultan K., Hajeer M.Y., Alhafi Z.M., Sukari T. (2025). Evaluation of the Relationship between Sagittal Skeletal Discrepancies and Maxillary Sinus Volume in Adults Using Cone-Beam Computed Tomography. Sci. Rep..

[24] Manson P.N., Ruas E.J., Iliff N.T. (1987). Deep Orbital Reconstruction for Correction of Post-Traumatic Enophthalmos. Clin. Plast. Surg..

[25] Gellrich N.-C., Rana M. (2018). Navigation and Computer-Assisted Craniomaxillofacial Surgery. Digital Technologies in Craniomaxillofacial Surgery.

[26] López-Silva F.A., Morales-Yépez H.A., Caballero-De-La-Peña U. (2024). Fundamentals of Intraoperative Navigation for Facial Fractures. Plastic and Reconstructive Surgery Fundamentals: A Case-Based and Comprehensive Review.

[27] Li B., Zhang L., Sun H., Yuan J., Shen S.G., Wang X. (2013). A Novel Method of Computer Aided Orthognathic Surgery Using Individual CAD/CAM Templates: A Combination of Osteotomy and Repositioning Guides. Br. J. Oral Maxillofac. Surg..

[28] Patel P.K., Zhao L., Morris D.E., Alves P.V. (2008). Our Experience with Virtual Craniomaxillofacial Surgery: Planning, Transference and Validation. Stud. Health Technol. Inform..

[29] Lu L., Wang H., Liu P., Liu R., Zhang J., Xie Y., Liu S., Huo T., Xie M., Wu X. (2022). Applications of Mixed Reality Technology in Orthopedics Surgery: A Pilot Study. Front. Bioeng. Biotechnol..

[30] Magalhães R., Oliveira A., Terroso D., Vilaça A., Veloso R., Marques A., Pereira J., Coelho L. (2024). Mixed Reality in the Operating Room: A Systematic Review. J. Med. Syst..

[31] Yamazaki A., Ito T., Sugimoto M., Yoshida S., Honda K., Kawashima Y., Fujikawa T., Fujii Y., Tsutsumi T. (2021). Patient-Specific Virtual and Mixed Reality for Immersive, Experiential Anatomy Education and for Surgical Planning in Temporal Bone Surgery. Auris. Nasus. Larynx.

[32] Ito T., Kawashima Y., Yamazaki A., Tsutsumi T. (2021). Application of a Virtual and Mixed Reality-Navigation System Using Commercially Available Devices to the Lateral Temporal Bone Resection. Ann. Med. Surg..

[33] Teatini A., Kumar R.P., Elle O.J., Wiig O. (2021). Mixed Reality as a Novel Tool for Diagnostic and Surgical Navigation in Orthopaedics. Int. J. Comput. Assist. Radiol. Surg..

[34] Stadnytskyi V., Ghammraoui B. (2024). Experimental Setup for Evaluating Depth Sensors in Augmented Reality Technologies Used in Medical Devices. Sensors.

[35] Pacheco-Barrios K., Ortega-Márquez J., Fregni F. (2024). Haptic Technology: Exploring Its Underexplored Clinical Applications—A Systematic Review. Biomedicines.

[36] Mangano F.G., Yang K.R., Lerner H., Admakin O., Mangano C. (2024). Artificial Intelligence and Mixed Reality for Dental Implant Planning: A Technical Note. Clin. Implant Dent. Relat. Res..

